# Impact of the SARS-COV-2 pandemic on access to health services in Angola: a focus on diagnosis and treatment services for tuberculosis

**DOI:** 10.3389/fpubh.2025.1530782

**Published:** 2025-04-24

**Authors:** Susanna Caminada, Roberto Benoni, Maria Grazia Dente, Claudia Robbiati, Joaquim Tomas, Giulia Natali, Luca De Simeis, Nsuka Da Silvia, Neusa Lazary, Paulo Siene Tienabe, Giovanni Putoto, Marianna Costanzo, Fabio Manenti, Maria Elena Tosti

**Affiliations:** ^1^Department of Public Health and Infectious Diseases, Sapienza University of Rome, Rome, Italy; ^2^National Center for Global Health, Italian National Institute of Health (Istituto Superiore di Sanità), Rome, Italy; ^3^Doctors with Africa CUAMM, Padua, Italy; ^4^Doctors with Africa CUAMM, Luanda, Angola; ^5^Department of Public Health, Bureau of Public Health of Luanda (Gabinete Provincial de Saúde de Luanda), Luanda, Angola; ^6^Programa Nacional de Controlo da Tuberculose (PNCT), Luanda, Angola

**Keywords:** tuberculosis, COVID-19, health services access, Angola, SARS - CoV-2

## Abstract

**Introduction:**

The SARS-CoV-2 pandemic had a profound impact on healthcare systems worldwide. In sub-Saharan Africa, it significantly affected several health services for infectious diseases such as HIV; however, less is known about its impact on Tuberculosis (TB). This study aimed to assess the pandemic’s impact on access to health services in Angola, focusing on diagnosis and treatment services for TB.

**Methods:**

An observational study combining data from routine statistics and surveys based on ad-hoc questionnaires was conducted on TB and non-TB services between 2018 and 2022. On routine data, temporal trends were analyzed comparing different non TB- and TB-specific indicators across the five-year period using the chi-square test. Questionnaires were administered to healthcare professionals from TB/non-TB services and structured interviews were conducted with TB patients to understand their perceptions about the impact of COVID-19 pandemic.

**Results:**

There was a significant decline in access to TB services during the pandemic, with a substantial decrease in reported cases (−15.5% in 2020; −18.3% in 2021) and treatment rate (from 86% in 2019 to 68% in 2020), an increase in multidrug-resistant-TB (from 0.2% in 2018 to 2.1% in 2022) and TB/HIV co-infections (from 6% in 2018 to 8.8% in 2021). The impact was most pronounced in the province of Luanda (capital city). TB services in Angola were disproportionately affected compared to general healthcare access indicators. The healthcare professionals’ and patients’ questionnaires showed that fear of COVID-19, unavailability of drugs, reduced income, and transportation challenges were the main barriers to healthcare access.

**Conclusion:**

The COVID-19 pandemic negatively impacted the TB services provision in Angola. This highlights the urgent need for health systems to develop robust contingency plans to ensure the continuity of TB services during and after public health crises and to maintain essential healthcare services by supporting the healthcare workforce and addressing barriers to patient access.

## Introduction

The COVID-19 pandemic and the measures undertaken to contain the spread of the SARS-CoV-2 virus led to an unprecedented global disruption of healthcare services (1–3).

The overwhelming impact of the pandemic on health systems across the world has brought attention to the vulnerabilities and fragilities of healthcare infrastructure, particularly in low- and middle-income countries. In these settings, already struggling health systems were further strained, posing significant challenges to the delivery of essential health services (4–6).

One critical area of healthcare that was severely affected by the pandemic was the delivery of prevention and treatment services for Tuberculosis (TB) (7). The redirection of healthcare resources and attention toward COVID-19, coupled with the limited capacity to simultaneously manage both TB and pandemic-related care, led to setbacks in TB diagnosis, treatment initiation, and patient follow-up (8, 9). Moreover, delays in healthcare-seeking behavior further exacerbated the situation, potentially contributing to increased TB transmission rates and poorer treatment outcomes (9, 10).

As a result, there was a significant global decline in the number of newly diagnosed and officially reported (i.e., notified) TB cases, along with an increase in the number of people with undiagnosed and untreated TB (11). The COVID-19 pandemic and its associated response also led to a substantial rise in projected TB mortality rates (12).

Angola, a country in southern Africa with an under-resourced health system and high levels of poverty (13), is among the 30 high-burden countries for TB and multidrug-resistant TB/rifampicin-resistant TB (MDR/RR-TB) (8). The COVID-19 pandemic significantly disrupted Angola’s healthcare services, with patients facing challenges in accessing medical facilities, disruptions in the supply of essential medications, and reductions in diagnostic and treatment services (14).

The present study was conducted as part of the *Project “CombaTB: Fighting TB and HIV at the times of Covid-19 through an increased access to prevention, early diagnosis, treatment and follow up services,”* financed by the Italian Agency for Development Cooperation and implemented by Doctors with Africa CUAMM in partnership with Luanda Health Office (Gabinete Provincial de Saúde de Luanda - GPSL) and the NGO Ajuda de Desenvolvimento de Povo para Povo (ADPP), with the technical support of the Italian National Institute of Health (15).

The primary objective of this study was to assess the impact of the COVID-19 pandemic on access to TB healthcare services in Angola over the period 2018–2022. Secondary objectives included exploring potential differences in service provision between the capital (Luanda) and other provinces, as well as investigating potential factors associated with limited access to TB healthcare services.

Given the substantial global impact of COVID-19 on TB control efforts, this research provides critical insights into the specific challenges faced in Angola and offers evidence to inform policy measures aimed at strengthening TB services and mitigating the setbacks caused by the pandemic. By understanding the extent of disruptions and the factors influencing service access, and by identifying barriers in TB diagnosis and treatment, this study can inform targeted interventions that enhance healthcare resilience and Angola, contributing to ongoing efforts to recover TB control progress and advance toward the End TB strategy’s targets (16).

## Methods

### Study design and ethical approval

This is an observational study using two sources of data: routine statistics and surveys based on ad-hoc questionnaires. The research was performed in accordance with the ethical standards of the 1964 Declaration of Helsinki. The study protocol received final approval from the Ethics Committee of the Republic of Angola Ministry of Health on February 1, 2023 (PARECER No. 005/C.E.M.S./2023).

### Setting and population

The study was conducted in Angola, a country located on the west coast of southern Africa with an estimated population of 34.1 million people in 2023. Angola is divided into 18 provinces and the capital city is Luanda.

In order to understand their perceptions about the impact of COVID-19 pandemic, two different questionnaires were administered to healthcare personnel and TB patients, respectively.

The sample of healthcare professionals included different profiles (e.g., doctors, nurses, laboratory technicians, pharmacists), employed in both TB-specific and non-TB specific primary level services. The sample included professionals from 8 facilities involved in the project within the province of Luanda.

Patients were recruited from the same 8 facilities based on year of TB diagnosis in order to compare the attitudes reported by patients diagnosed in 2019 with those in 2020–2021 and 2022. Inclusion criteria for patients were as follows: patients with a TB diagnosis at one of the 8 health facilities in the province of Luanda, with a completed treatment and outcome or dropouts between 2019 and 2022, aged18 years or older.

### Data source for routine statistics

To assess and quantify the impact of the COVID-19 pandemic on access to healthcare services, routine data collection on TB-specific and non-TB-specific services was analyzed. Data on other health conditions were also acquired to determine whether the pandemic’s impact was specific to TB or affected healthcare access more broadly. Data for 2018–2022 have been considered and collected at an annual level of aggregation. The period was chosen to assess the trend per year in the period before and during the COVID-19 pandemic, until one year after the pandemic.

With regard to TB services, data were collected on two different levels: at the national level and the level of the Province of Luanda. Non-TB indicators, on the other hand, were only collected at the provincial level.

Data sources were the Programa Nacional de Controlo da Tuberculose (PNCT), which provided data for the years 2018 to 2022, disaggregated by province used for TB indicators, and Gabinete Provincial de Saùde de Luanda (GPSL) for other indicators (non-TB).

### Selected indicators

Evaluated TB-specific indicators were: number of new diagnoses, number of relapses, number of MDR-TB cases, number of HIV/TB co-infections, number of deaths due to TB, number of patients started on TB treatment.

Selected treatment outcomes were: number of treatment successes, number of cases that completed treatment, number of dropouts, number of treatment failures, number of patients not evaluated.

Relapse was defined as a recurrent episode of tuberculosis caused by the same strain as was identified at baseline. Treatment success was defined as a patient whose treatment outcome is either cured or completed. Dropout was defined as a patient who did not start treatment or whose treatment was interrupted for two or more consecutive months. Treatment failure was defined as a patient whose anti-tuberculosis treatment regimen needed to be terminated or permanently changed to a new regimen or treatment strategy (17). Non-TB indicators of access to health services evaluated were: antenatal visits, deliveries within health facilities, vaccinations, and hospital admissions.

### Surveys and data collection

Two ad-hoc standardized questionnaires were developed by a multidisciplinary team, based on the available literature, to explore possible factors associated with reduced access to TB healthcare services.

The first questionnaire was designed for healthcare professionals and was self-administered after explanation by a project operator. Its primary objective was to assess healthcare staff perceptions regarding the impact of COVID-19 on access to healthcare facilities in Angola. The questionnaire included sections on socio-demographic information and healthcare professionals’ perspective on how the pandemic affected both patients and the healthcare system (Supplementary Annex A).

The second questionnaire (Supplementary Annex B) was administered to TB patients from different facilities through interviews conducted by appropriately trained interviewers from the project partner *Ajda de Desenvolvimento de Povo para Povo Angola* (ADPP). These interviews aimed to investigate possible personal determinants influencing variations in access to healthcare services, such as socio-economic conditions, distance to health facilities, and fear of stigma and discrimination. The questionnaire collected sociodemographic data, basic information on the disease (localisation, bacilloscopy and HIV testing, treatment outcome), and included specific questions on attitudes during COVID-19 pandemic, which were administered only to patients recruited in 2020 and 2021. Patients were recruited based on the records of the 8 different healthcare facilities involved. The operators in charge contacted the selected patients to confirm their eligibility and willingness to take part in the study. To reduce costs and increase the feasibility of the study, the interviews were conducted via telephone.

Data were collected from 10 February to 15 March 2023. Interviews with patients and healthcare personnel were conducted after informed consent was obtained, and the interviewee was explained the lines of conduct of the study and its purposes.

### Statistical analysis

Descriptive statistics of the items included in the questionnaires were performed. Continuous variables are expressed as medians [range] and categorical variables are expressed as proportions. The following possible univariate associations were explored using the chi-square test and Fisher’s exact test, where appropriate, for categorical variables and the Mann–Whitney or Kruskal-Wallis non-parametric tests for continuous variables: trend in new diagnosis, MDR-TB, HIV coinfection, treated patients, by year; trend in access to non-TB services, by year; opinion personnel on impact of the pandemic in TB and non-TB services; between sociodemographic characteristics and opinion on COVID-19 pandemic impact and type of patients (TB vs. not TB or TB treated vs. lost to follow-up). A *p*-value <0.05 was considered statistically significant. All analyses were performed with STATA 16 (StataCorp LLC, 4905 Lakeway Drive, College Station, TX, USA).

## Results

### Access to TB prevention and treatment services

During the observation period (2018–2022), the number of TB cases notified annually in Angola to the PNCT ranged approximately between almost 77,000 and 63,000.

**Figure 1:**
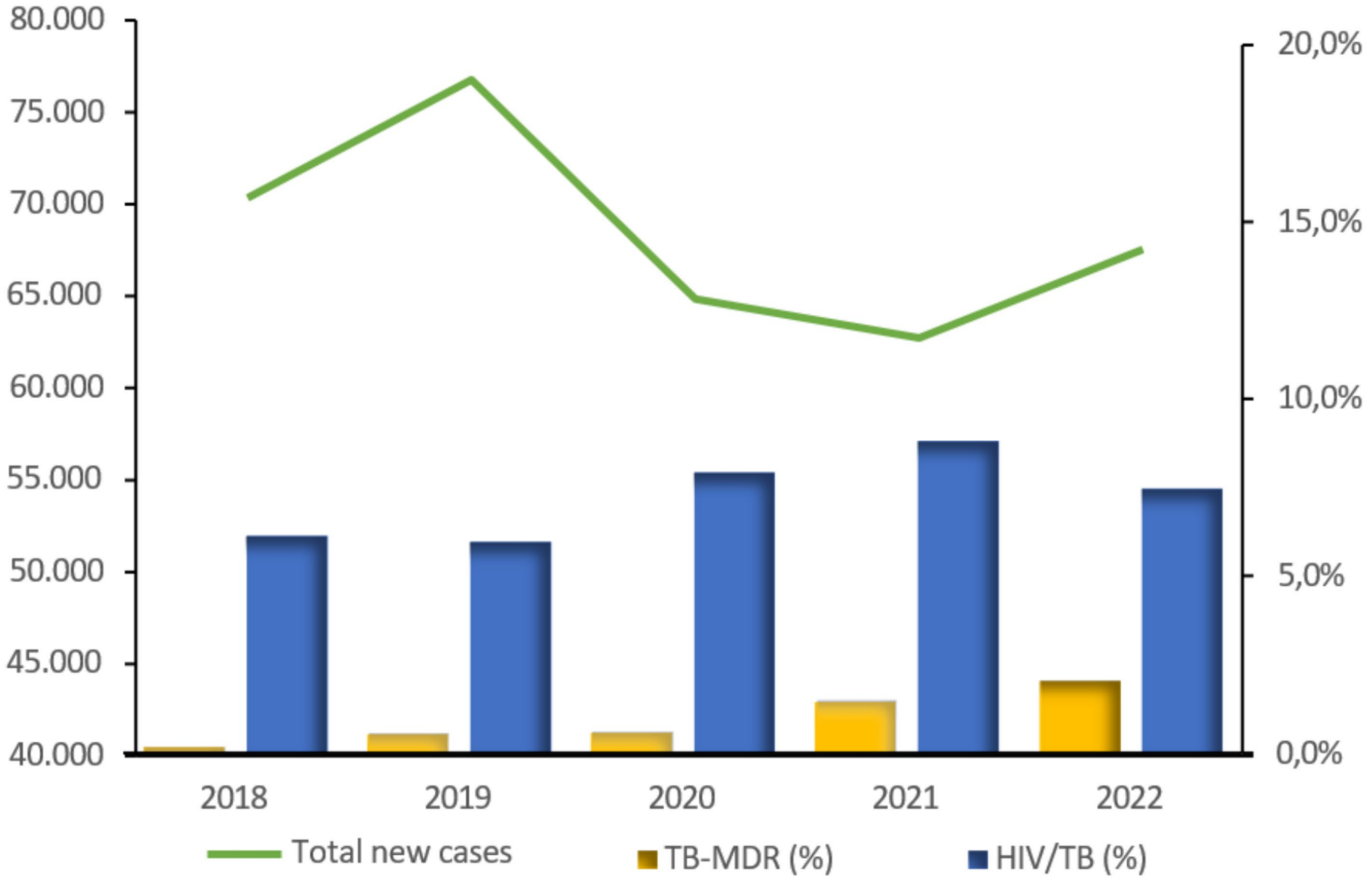
Trend by year of total TB cases and in the percentages of MDR and HIV/TB coinfected cases notified in Angola. Source: PNCT.

The yearly trend of notified cases shows an initial increase in reports between 2018 and 2019, followed by a significant decrease in 2020 and 2021 (Figure 1). Specifically, the number of cases dropped from around 77,000 in 2019, to 65,000 in 2020, representing a reduction of 15.5 percent; a further decrease is observed in 2021 (−18.3 percent compared to 2019). Although reporting partially recovered in 2022, the number of notified cases remained 12.0% lower than in 2019.

Figure 1 also shows the trend in the percentages, among cases diagnosed by year, of multidrug-resistant forms of TB (TB-MDR) and HIV co-infected cases (HIV/TB). Regarding the TB-MDR percentage, the trend from 2018 to 2022 has been steadily increasing; in 2022, 2.1% of new diagnoses were TB-MDR, a tenfold increase from 0.2% in 2018. In absolute numbers, TB-MDR cases nearly tripled between 2020 and 2021; this increasing trend is still present in 2022 (*p* < 0.001). The percentage of HIV-coinfected patients remained stable in 2018 and 2019 but increased in the pandemic biennium, from about 6% in 2018 and 2019 to 7.9% in 2020 and 8.8% in 2021 (<0.001). In absolute numbers, almost 1,800 more co-infected cases were recorded in the biennium 2020 and 2021 than expected, based on what was recorded in the pre-pandemic years 2018 and 2019.

**Figure 2:**
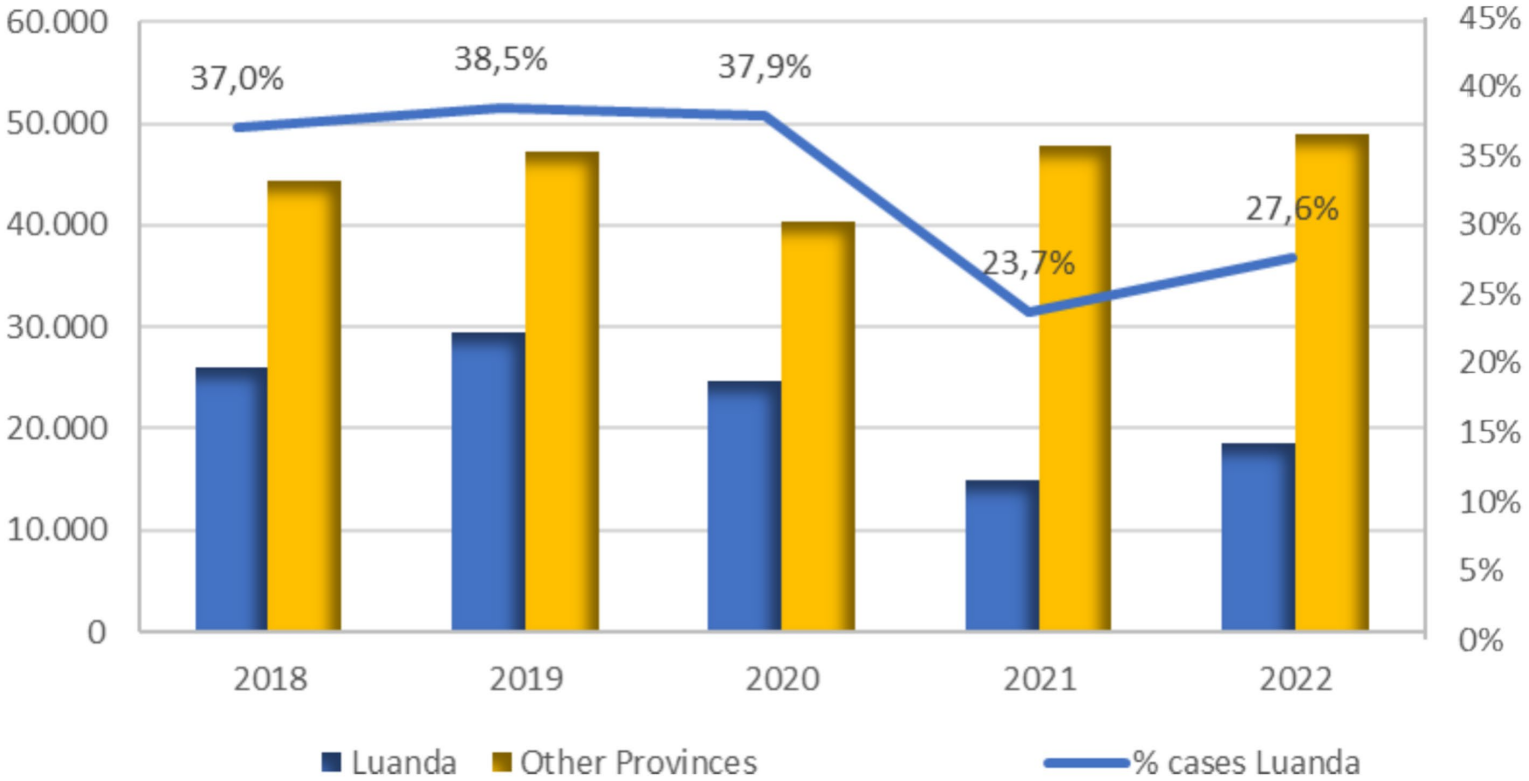
Trends by year in the absolute number of new cases in Luanda province and the remaining provinces, and the percentage of Luanda province cases compared to the national figure. Source: PNCT.

Figure 2 shows a comparison of annual new cases diagnosed in Luanda and in all other provinces; the trend shows a significant decline in new cases during the pandemic especially in the province of Luanda; such a decrease began as early as 2020 and became more evident in 2021. Compared to the about 29,500 cases reported in 2019 in Luanda, these drop to about 14,500 in 2021. A slight upswing in new diagnoses is observed in Luanda in 2022 (18,000 cases). A very different trend is shown by the newly diagnosed cases in the other provinces, where the decrease of cases during the pandemic was much more limited and affected only 2020, while in 2021 and 2022 new cases are slightly increasing even compared to 2019. A consequence of these trends is the decrease in the % of cases diagnosed in Luanda province: this was 39% in 2019 and drops to 24% in 2021.

Over the observation period, Luanda consistently recorded a higher percentage of TB-MDR and HIV/TB co-infected cases compared to other provinces. Moreover, increasing trends were particularly evident in Luanda, especially for TB-MDR cases.

**Figure 3:**
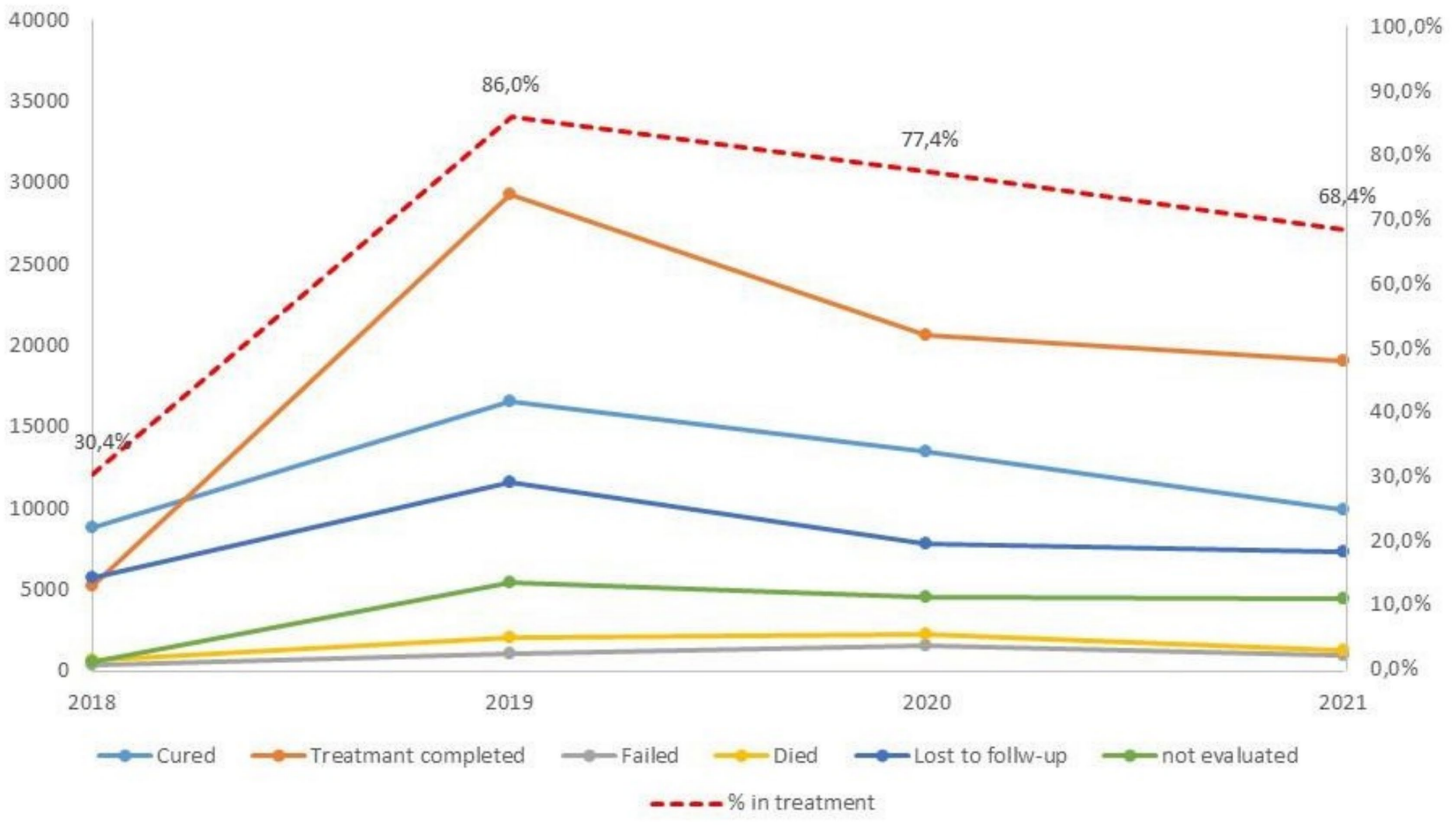
Trends by year in the number of patients treated by TB treatment outcome and of the percentage of patients started on treatment for TB. Source: PNCT.

Figure 3 shows the trends by year in the percentage of patients started on treatment for TB and the distribution of patients by treatment outcome (absolute numbers); the percentage of patients receiving treatment, which was very low in 2018, increased to over 86 percent of diagnoses in 2019 and then declined again during the pandemic years (68% in 2021).

The decrease in treatments observed between 2020 and 2021 seems to mainly affect all treatment successes, i.e., cured patients (negative bacilloscopy) or complete treatments (without performing bacilloscopy).

The decreasing trend by year in the percentage of patients started on treatment for TB is particularly evident in Luanda where in comparison with the 87.6% of treated in 2019, they are 63.2% in 2020 and 41.6% in 2021 (*p* < 0.001). The effect of pandemic in the other provinces is lower and it seems to be present only in 2021 (76.8% in 2021 vs. 85.0% in 2019) (Supplementary Figure 2).

The pandemic years also see an increase in relapses among new cases, that is, patients who become bacteriologically positive after being treated for TB and declared cured or having completed treatment: a percentage increase of 20.2% was observed between the absolute value of 2019 and 2020, 22.1% between 2020 and 2021, but only 4% between 2021 and 2022.

### Access to other health services (not dedicated to TB)

Regarding access to non-TB services, no clear trends were observed during the pandemic biennium. Some indicators, such as deliveries, first antenatal visits, and vaccinations, show decreasing values already in 2019, and a progressive increase between 2020 and 2021, followed by another decrease in 2022. The number of hospitalizations by year shows a slightly upward trend throughout the observation period; the only indicator that shows a drop in 2020 is the number of “Ambulatory visits” (Supplementary Figure 3).

### Interviews with healthcare personnel

In total, 57 health workers were interviewed, and all healthcare facilities are reasonably equally represented in the TB and non-TB fields. Table 1 describes the selected sample according to TB or non-TB service affiliation.

**Table 1:** Descriptive table of health personnel by field of activity (TB/non-TB) and opinions on the impact of the pandemic on access to services and on their work.

	TB (*n*° = 31)	Non-TB (*n*° = 26)	Total (*n*° = 57)	*p*-value
**Description of health personnel**				
Gender				
Male	7 (22.6)	6 (23.1)	13 (22.8)	0.605
Female	24 (77.4)	20 (76.9)	44 (77.2)	
Median age (range)	47 (27–53)	44 (25–66)	45.5 (25–66)	0.850
Profession				
Medical doctor	5 (16.1)	3 (11.5)	8 (14.0)	**0.016**
Nurse	15 (48.4)	22 (84.6)	37 (64.9)	
Laboratory technician	8 (25.8)	1 (3.8)	9 (15.8)	
Other	3 (9.7)	0 (0.0)	3 (5.3)	
**Impact of the pandemic on access to services**				
Have you noticed a decrease in patient attendance?				
No	15 (48.4%)	5 (19.2%)	20 (35.1%)	**0.041**
Yes	15 (48.4%)	17 (65.4%)	32 (56.1%)	
I do not know	1 (3.2%)	4 (15.4%)	5 (8.8%)	
The patients who presented had more serious conditions than before the pandemic?				
No	8 (32.0%)	6 (28.6%)	14 (30.4%)	0.227
Yes	12 (48.0%)	6 (28.6%)	18 (39.2%)	
I do not know	5 (20.0%)	9 (42.8%)	14 (30.4%)	
**Impact of the pandemic on health personnel work**				
Has there been any change in the number of health personnel employed?				
No	4 (12.9%)	3 (11.5%)	7 (12.3%)	>0.999
Yes	25 (80.6%)	21 (80.8%)	46 (80.7%)	
I do not know	2 (6.5%)	2 (7.7%)	4 (7.0%)	
If yes, how?				
Decreased	18 (85.7%)	11 (84.6%)	29 (85.3%)	>0.999
Increased	3 (14.3%)	2 (15.4%)	5 (14.7%)	
Have there been any changes in staff shifts?				
No	2 (6.5%)	5 (19.2%)	7 (12.3%)	0.160
Yes	29 (93.5%)	20 (76.9%)	49 (86.0%)	
I do not know	0 (0%)	1 (3.9%)	1 (1.7%)	
If yes, how?				
Longer	10 (41.7%)	7 (53.8%)	17 (45.9%)	0.512
Shorter	14 (58.3%)	6 (46.2%)	20 (54.1%)	
Have the opening hours to the public been limited compared to normal?				
No	11 (35.5%)	13 (50.0%)	24 (42.1%)	0.224
Yes	20 (64.5%)	12 (46.1%)	32 (56.1%)	
I do not know	0 (0%)	1 (3.9%)	1 (1.8%)	

Bold values are those that are statistically significant (*p*-value < 0.05).

In general, the majority of respondents were female (77%), and the median age was 45.5 years (range 25–66). Nurses were the most represented profession, accounting for 65% of the sample. No significant differences was observed between the two groups except in the qualification; in fact, in the non-TB area, nurses represented about 85% of the sample (*p* = 0.016).

Table 1 also shows the perception of health workers on the impact of the pandemic on patients and on staff workload. Regarding the impact of the pandemic on patients’ access to services, 56% of respondents observed a decrease in access to the service where they work. The percentage was significantly higher (*p* = 0.041) in the group of non-TB service workers. Regarding the clinical conditions of patients during the pandemic, 40% of health workers stated that more severe patients were accessing services than before the pandemic, compared to 30% who disagreed and 30% who were uncertain. Among TB staff, the percentage who reported more severe patients during the pandemic was higher (48% vs. 29%), but not statistically significant.

Among the reasons for a decrease in access, respondents identified (multiple answers possible) fear of contracting the virus (46%), fear of being tested for SARS-CoV-2 and being locked up in special facilities in the event of a positive test result (40%), difficulty in reaching the facilities due to lack of public transport (30%), followed by the worsening economic situation preventing access to services (19%). Lesser-cited reasons included the reduced opening hours of healthcare services (14%), the awareness of the decreasing number of health personnel and of the health services available (11%), or the awareness of the lack of necessary treatments for different medical conditions (4%). No statistically significant differences were observed in the responses on possible reasons for the reduction in access between TB and non-TB service healthcare workers.

The decrease in the number of accesses to health facilities, according to the operators, affected both sexes (according to 66% of respondents). Considering the age groups, most respondents (58%) did not think the impact was equally distributed; 51% of respondents claimed it mainly affected children. This was found both in TB and non-TB facilities.

Over 80% of respondents stated that the pandemic has caused variations in both the number and shifts of staff employed; in particular, according to more than 85% of the respondents, staffing levels have decreased (with no statistically significant differences observed between TB and non-TB areas). As far as shifts are concerned, these appear to have varied more in the TB wards, but the direction of the variation is unclear, as 46% of the operators belived they are longer, while 54% belived they are shorter. No clear perception of a change in opening hours was observed among operators (Table 1).

When asked to quantify from 1 to 10 how much the pandemic has changed their work, the median response was ‘7’, with a range of 1 to 10. The majority (63%) of the respondents reported receiving no additional compensation for the increased workload. More than 96% of the operators believed that in 2022 the situation went back to normal, with no significant differences between TB and non-TB respondents.

Health operators’ questionnaire included also questions on a more general impact of COVID-19 in Angola (data not shown). Most operators (78%) believed that the pandemic affected Angola less than other African countries, while 15% believed the impact was comparable to other African nations. Only a few operators (5%) responded that the impact of the pandemic in Angola was similar to the rest of the world or more remarkable than in the rest of Africa.

The most commonly cited reasons for Angola’s relatively lower impact were the hot climate of the country (65%) and the strict containment measures (42%), followed by the young age of the population (12%). Fewer, on the other hand, mentioned the issue of under-reporting of cases (7%) and the habit of gathering outdoors (5%).

### Interviews with TB patients

The study protocol involved the enrolment of 160 patients. To reach the expected number, 412 patients (36 to 60 per facility) were selected from the facilities’ registers; however, 34% (142 patients) were unreachable due to a missing or incorrect telephone number. Of the remaining 270 patients contacted by telephone, 62% accepted the interview (acceptance rate ranging from 44 to 91% across different facilities).

A total of 168 patients were interviewed, ranging from 19 to 23 per healthcare facility. Among them, 81 (48%) had a known treatment outcome (cured or successfully treated) and 87 patients (52%) had dropped out of treatment and follow-up. The recruitment method ensured a balanced number of patients with known outcomes and those lost to follow-up each year, and the distribution of loss to follow-up was not significantly different in the different periods 2019, 2020–2021 and 2022 (*p* = 0.988).

Patients generally were young (median age 31, range 20–56), with a predominance of men (67.9%). 31.5% of surveyed patients were unemployed. The patients’ families comprised five members (median value), although only 73% reported having a reference person to support them in managing the disease. From the point of view of disease characteristics, almost all patients were first diagnosed with TB (97%) and had a pulmonary localization (96%). No significant differences were observed in patient demographics or disease characteristics across the three study periods.

Among the services indicative of access to essential TB services, we considered performing bacilloscopy and HIV testing, as these are standard guidelines for all TB patients. Overall, only 86% of cases with pulmonary localization were microbiologically diagnosed using bacilloscopy, and only about 76% of patients were tested for HIV antibodies. Encouragingly, in 2022, 97% of all cases (pulmonary and extra- pulmonary) underwent bacilloscopy, compared to 82% in 2019 and 79% in 2020–2021 (*p* = 0.039).

A comparison between patients lost to follow-up and patients treated/cured showed that the majority of patients lost at follow-up were men, 74.7%, compared to 60.5% of treated/cured patients (*p* = 0.049), and younger: median age of 29 [range 18–48], compared to treated/cured patients, with a median age of 32 [18–59] (*p* = 0.007). In contrast, no statistically significant differences in educational attainment were observed between the two groups. Forty percent of patients lost to follow-up were unemployed, compared to 22% of treated/cured patients (data not shown).

Considering the 3 periods separately (2019, 2020–2021, 2022), almost no significant differences were found in patient characteristics or disease-related factors that may have been determinants of loss to follow-up rather than stay in treatment. The only notable difference was in employment status, in 2019, and in age in 2022 (Supplementary Table 1). Some conditions (use of public transport, concern of infections in public transport or in health facilities and reliance on private pharmacies), potentially linked to the treatment outcome, were not associated, over the three periods (pre-pandemic, pandemic, post-pandemic).

In addition to the conditions presented, the possible influence of the distance between the patient’s home and the health facility at which they were being treated was also assessed. Again, no significant differences were found between patients cured/treated and those lost to follow-up. Interestingly, among the patients recruited in 2019 in the pre-COVID-19 era, infectious diseases of concern (e.g., for contagion in public transport or health facilities) included influenza, malaria, leprosy, and HIV. Even in the years after 2020, COVID-19 was not the only concern.

Table 2 focuses on conditions potentially associated with the likelihood of TB patients dropping out of treatment in the pandemic years (2020 and 2021) and post-pandemic period (2022), also analysing the impact on patients’ income. Almost all the considered conditions, potentially affecting treatment outcome, were reported more frequently during the pandemic years (2020–2021) than in 2022, without significant differences between cured patients and lost to follow-up. Notably, a high percentage of respondents reported a reduced income due to the pandemic in 2020–2021 (61 and 69%). These percentages were lower in both groups in 2022 (56 and 44%).

**Table 2:** Specific pandemic and post-pandemic conditions potentially associated with the likelihood of TB patients dropping out of treatment and impact of the pandemic on their income, by period (2020–2021 vs 2022) and treatment outcome.

Conditions potentially affecting treatment outcome	2020–2021			2022		
	*N*° (%)	*N*° (%)	*p*-value	*N*° (%)	*N*° (%)	*p*-value
	Treated	Lost		Treated	Lost	
Difficult in reaching the facility	14 (51.9)	15 (50.0)	0.889	7 (50.0)	4 (26.7)	0.264
Fear of SARS-CoV-2 testing	20 (71.4)	18 (60.0)	0.360	5 (33.3)	5 (33.3)	>0.999
Fear of a positive test for SARS-CoV-2	22 (78.6)	17 (56.7)	0.076	8 (53.3)	7 (43.3)	0.594
Fear of social isolation	16 (57.1)	21 (70.0)	0.309	7 (50.0)	7 (46.7)	0.858
Known lack of staff	10 (35.7)	10 (35.7)	>0.999	6 (37.5)	5 (31.2)	0.710
Known unavailability of drugs	14 (50.0)	14 (50.0)	>0.999	4 (25.00)	8 (50.0)	0.144
Reduced income due to the pandemic	17 (60.7)	20 (69.0)	0.514	9 (56.3)	7 (43.7)	0.480

## Discussion

The results of this study show that the COVID-19 pandemic negatively impacted the access to TB services in Angola. A decrease in new TB cases was registered during the pandemic, along with a downward trend in the number of treated patients and treatment successes and an increase in patients with MDR-TB and HIV/TB co-infection. The substantial reduction in notified TB cases during 2020 and 2021 likely resulted from the healthcare system strains, COVID-19 restrictions, and patient hesitancy to seek care, rather than an actual drop in infections (8). The partial recovery of TB cases in 2022 does not bring the reported cases back to pre-pandemic levels, indicating the need for sustained efforts to restore and maintain TB services.

The capital, Luanda, appears to have experienced the most significant impact, characterized by a substantial decline in newly reported cases and a notable increase in HIV/TB patients - trends less pronounced in the other provinces. In general, a large proportion of TB cases in Angola are diagnosed in the province of Luanda: although its population currently accounts for about 23% of Angola’s total population, between 25 and 39% of the total national cases were reported in the province of Luanda during the study period. This uneven distribution of cases across different provinces emphasizes the need for targeted strategies to address regional disparities. Several factors contribute to making Luanda Angola’s TB epicentre, including its population density, overcrowded living conditions due to its rapid urbanization, the presence of migrants and transient populations, its healthcare infrastructure and the higher burden of HIV (18).

The increasing trend in TB-MDR cases shown in the results is alarming and requires immediate attention. This spike in multidrug-resistant cases, with a tenfold increase from 2018 to 2022, suggests that COVID-19 exacerbated challenges in diagnosis, treatment, and drug management of TB cases. Urgent measures are required to address this growing issue, especially in the capital city (18).

Additionally, the data reveals increased TB/HIV co-infected cases during the pandemic years. Thus, the pandemic seems to have affected not only the services strictly related to TB but also those integrated with it such as those for HIV (19). This finding highlights the importance of integrated healthcare services to effectively manage both conditions. Specialized programs and resources may be needed to address this complex public health challenge (7).

The decline in the number of TB patients receiving treatment, particularly in 2021, correlates with a noticeable increase in relapse cases, suggesting an impact of COVID-19 also on follow-up and treatment completion. In contrast, the low number of TB patients being treated for tuberculosis in 2018 shown in the results is plausibly attributable to a drug shortage that affected the country in 2017, which was then resolved during 2018.

The data on access to other healthcare services at the national level shows a somewhat different trend for the pandemic two-year period. In fact, the only indicator showing a clear decline in 2020 is the number of Outpatient visits. Instead, some of the indicators considered (deliveries, prenatal visits, vaccinations) showed decreasing values already in 2019. One explanation may be that the reporting of information for 2019 was collected/assembled during 2020 and may therefore have been impacted by the pandemic due to the unexpected workload and difficult working conditions faced by healthcare workers in the early phases of the emergency. However, the fact that the impact of COVID-19 does not seem to occur in such a pronounced manner for these services also confirms that TB services seem to be more vulnerable and ‘sacrificed’ in times of public health crises, together with Outpatient visits that are presumably deprioritized as considered non-urgent in many cases. Staff interviews do not seem to provide any clear explanations for this phenomenon. Interestingly, in contrast to the data from the routine statistics, the perceived impact on access to services is lower among staff working in TB facilities than in non-TB facilities. However, although the sample size of 57 healthcare workers was reasonably balanced between TB and non-TB service areas, non-TB services had a higher proportion of nurses in their workforce, indicating potential staffing differences by professional category between service types.

Among those who reported a decrease in access, 40% noted that more severe patients were accessing services than before, highlighting the challenges in maintaining routine healthcare during a pandemic (3).

In general, in the opinion of surveyed healthcare workers, the primary reasons for the decrease in patient flow during the pandemic were the fear of being tested for SARS-CoV-2 (40%) and the fear of testing positive (46%), followed by the shortage of public transportation and the worsening economic conditions. These challenges underscore the need for robust public health measures and strategies to ensure that patients can continue to access healthcare safely. Such strategies must extend beyond the health sector alone.

According to the operators, the decrease in access disproportionately affected children, both in TB and non-TB facilities - consistently with other studies that have shown a marked reduction in TB notification rate among the paediatric population (20).

A significant proportion of responding healthcare workers (81%) reported a decrease in the number of staff employed during the pandemic, with no notable differences between TB and non-TB areas. Despite this, the majority (63%) did not receive compensation for the increased workload. Staff shortages have been a significant hallmark of the COVID-19 pandemic, caused both by staff isolation/quarantine and reassignments to COVID- related services (7).

The results of the questionnaires for health personnel in TB centres align with what has been described in previous qualitative studies conducted in other countries in Africa (21, 22).

Interviews with TB patients investigated the possible reasons that could explain the reduced access to services during the pandemic: fear of social isolation due to possible SARS-CoV-2 infection, non-availability of drugs in facilities and reduced income due to the pandemic, as well as difficulties in reaching facilities.

About 52% of the interviewed patients had dropped out of treatment and follow-up. The decision to recruit a balanced number of patients, both those with known outcomes and those lost to follow-up, was driven by the intention not to primarily evaluate the pandemic period’s loss proportion compared to the previous one, already explored by the routine statistics, but to delve into the underlying reasons for it. This group tended to be younger and comprised a higher percentage of unemployed individuals. Comprehending the characteristics of this specific subgroup is important for designing targeted interventions to improve adherence and retention in TB treatment programs. The crucial aspect lies in recognizing and directing attention towards high-risk populations at an elevated risk of discontinuing treatment. Tailored interventions and support for these groups can in fact help improve treatment outcomes.

The study also examined factors potentially associated with TB treatment outcomes and assessed the influence of conditions such as distance to the healthcare facility, concern about infectious diseases, and modes of transportation. Surprisingly, no significant differences were found between patients with positive treatment outcomes and those who were lost to follow-up across the reporting periods. This lack of notable differences underscores the complex, multifaceted factors influencing treatment adherence. This is partly consistent with previous studies that tried to assess factors associated with loss to follow-up in TB treatment in some specific contexts of Angola (23) and other African countries (24).

Specific questions regarding the impact of the COVID-19 pandemic on TB treatment outcomes were asked to patients enrolled in 2020, 2021, and 2022. In 2021, the second year of the pandemic, certain factors, such as fear of social isolation following possible SARS-CoV-2 infection, not seeking treatment when drugs were unavailable, and reduced income due to the pandemic, were more prevalent among patients who did not complete treatment. This suggests that the pandemic may have had a notable impact on the adherence and retention to treatment of TB patients. It is interesting to note the high number of people who reported a loss of income due to the pandemic, especially in 2021.

Moreover, the data indicates variations in access to crucial TB services such as bacilloscopy and HIV testing. While the 2022 data shows improvements, the overall testing rates remain suboptimal, which may hinder early diagnosis and timely treatment initiation. However, it should be noted that this information was collected through the examination of medical records, so it is possible that some of the information may not have been correctly documented and some of the tests (bacilloscopy and HIV) that appear to be unreported were instead performed.

The findings of this operational research highlight the need for tailored strategies to maintain essential healthcare services, support the workforce, and address barriers to patient access during health emergencies. The impact of the COVID-19 pandemic on TB care outcomes underscores the need for health systems to develop robust contingency plans for maintaining essential TB services during health emergencies (1). This includes developing strategies to address patient fears and financial challenges during pandemics: maintaining public trust in the health system’s ability to safely meet essential needs and to control infection risk in health facilities is critical to ensuring appropriate care-seeking behavior and adherence to public health advice (2).

## Study limitations and strengths

This operational research has some limitations that should be considered when interpreting the results.

The main limitation of the first part of the study, based on routine statistics, is that the accuracy and reliability of the data used in the analysis depend on the quality of routine data collection. Although using routine data is usually convenient, cost-effective, and time-efficient, it should be considered that any issues with data accuracy, completeness, or consistency could have led to biased results. Also, the study primarily relies on annual aggregated data, which may obscure significant temporal trends and variations. Limited data disaggregation may also hinder the ability to identify subpopulations or areas disproportionately affected by the pandemic. There may also be data gaps for other relevant variables that could impact access to health services that were not considered. However, including non-TB health service indicators allows for a comparative assessment, helping determine whether the effects of the pandemic on health service access are specific to TB services or more widespread.

Regarding the second part of the study, based on surveys, there could have been a sampling bias since the sample of healthcare professionals and patients may not be fully representative. The sample size of healthcare professionals and patients are relatively small, which could limit the statistical power and the ability to detect significant associations or differences. Moreover, self-reported data from both healthcare professionals and patients can lead to recall bias and response bias (e.g., social desirability bias); for instance, patients may be hesitant to express negative opinions or experiences due to concerns about stigma or discrimination, which may particularly influence responses from those who were lost to follow-up.

Another factor that could have led to a biased selection is the fact that 34% of patients were not reachable due to a missing or incorrect telephone number: these patients could have had different attitudes in comparison with the interviewed patients.

Nevertheless, the study includes data from multiple time points (2019, 2020–2021, and 2022) to assess changes over time. This longitudinal approach allows for the examination of trends and the potential impact of COVID-19 over several years - although it should be kept in mind that the pandemic’s consequences may have gone even beyond 2022. Lastly, a final strength lies in the study being done in partnership with NGOs (including local organizations) and other entities with deep knowledge of the region and strong community connections. This was crucial for understanding some of the responses and interpreting the results, but also to obtain interviewees’ cooperation.

In conclusion, this research offers valuable insights and essential takeaways for future epidemic and pandemic preparedness. Furthermore, as we move from over three years of a pandemic marked by recurring surges to an endemic phase, it becomes imperative for all systems to adapt to this new reality.

## Data Availability

The raw data supporting the conclusions of this article will be made available by the authors, upon reasonable request.
