# Supplementary material for: Impact of the SARS-COV-2 pandemic on access to health services in Angola: a focus on diagnosis and treatment services for tuberculosis
Source: Front Public Health. 2025 Apr 24;13:1530782. doi: 10.3389/fpubh.2025.1530782 (PMC12058889; doi:10.3389/fpubh.2025.1530782)
Supplement: Supplementary file 1 [file Data_Sheet_1.docx]

**Supplementary materials**

**Supplementary Figure 1. Trends by year in the percentage of TB-MDR and HIV/TB case rates. Comparison between Luanda province and other provinces. Source: PNCT**


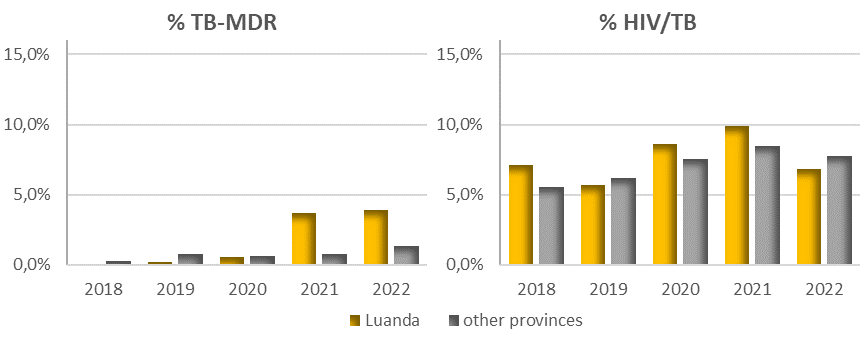


**Supplementary Figure 2. Trends by year in the percentage of patients started on treatment for TB in Luanda and i the Other Provinces. Source: PNCT**


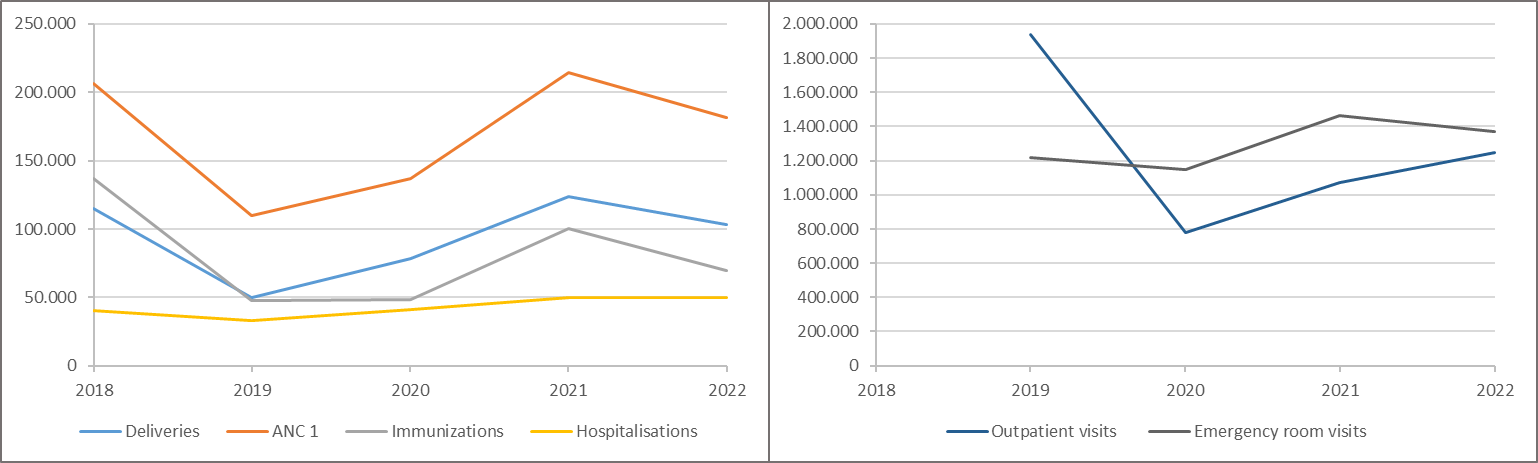
**Supplementary Figure 3. Trend by year of access to health facilities for deliveries, first antenatal visit (ANC 1), vaccinations, and** **hospitalizations. Source: Gabinete Provincial de Saùde de Luanda. 2018-2022***

ANC 1: First antenatal visit

* Data of Outpatient visits and Emergency room visits are not available for 2018

**Supplementary Table 1. Descriptive table of interviewed patients by reporting period and treatment outcome**

|  | **2019** | | | **2020-2021** | | | **2022** | | |
| --- | --- | --- | --- | --- | --- | --- | --- | --- | --- |
|  | **known outcome** | **Lost** | **p-value** | **known outcome** | **Lost** | **p-value** | **known outcome** | **Lost** | **p-value** |
| **Socio-demographic characteristics** | **N° (%)** | **N° (%)** |  | **N° (%)** | **N° (%)** |  | **N° (%)** | **N° (%)** |  |
| Median age (range) | 32 (18-59) | 30 (18-47) | 0.231 | 30.5 (18-48) | 29 (18-45) | 0.336 | 39 (23-56) | 27 (20-48) | **0.006** |
| Gender |  |  |  |  |  |  |  |  |  |
| Male | 22 (59.5) | 29 (74.4) | 0.167 | 16 (57.1) | 23 (76.7) | 0.113 | 11 (68.8) | 13 (72.2) | >0.999 |
| Female | 15 (40.5) | 10 (25.6) |  | 12 (42.9) | 7 (23.3) |  | 5 (31.2) | 5 (27.8) |  |
| Educational level |  |  |  |  |  |  |  |  |  |
| Low education | 12 (32.4) | 14 (35.9) | 0.750 | 11 (39.3) | 15 (50.0) | 0.412 | 7 (43.7) | 9 (50.0) | 0.744 |
| High education | 25 (67.6) | 25 (64.1) |  | 17 (60.7) | 15 (50.0) |  | 9 (56.3) | 9 (50.0) |  |
| Profession |  |  |  |  |  |  |  |  |  |
| Student | 0 (0.0) | 1 (2.6) | **0.033** | 3 (10.7) | 0 (0.0) | 0.185 | 1 (6.2) | 1 (5.6) | 0.978 |
| Craftsman/special. labourer/farmer | 7 (18.9) | 5 (12.8) |  | 6 (21.4) | 3 (10.0) |  | 3 (18.7) | 3 (16.7) |  |
| Qualified profess. /services | 13 (35.1) | 6 (15.4) |  | 4 (14.3) | 8 (26.7) |  | 7 (43.8) | 7 (38.9) |  |
| Other/ Occasional worker | 10 (27.0) | 8 (20.8) |  | 7 (25.0) | 6 (20.0) |  | 2 (12.5) | 4 (22.2) |  |
| Unemployed | 7 (18.9) | 19 (48.7) |  | 8 (28.6) | 13 (43.3) |  | 3 (18.7) | 3 (16.7) |  |
| Median N° of cohabitants (range) | 5 (1-15) | 5 (1-9) | 0.560 | 6.5 (2-12) | 5 (0-12) | 0.139 | 5 (1-10) | 5 (1-12) | 0.972 |
| **Disease characteristics** |  |  |  |  |  |  |  |  |  |
| Case classification |  |  |  |  |  |  |  |  |  |
| New | 37 (100.0) | 37 (97.4) | >0.999 | 26 (92.9) | 29 (96.7) | 0.737 | 16 (100.0) | 17 (94.4) | >0.999 |
| Already treated | 0 (0.0) | 1 (2.6) |  | 1 (3.6) | 0 (0.0) |  | 0 (0.0) | 1 (5.6) |  |
| Transferred | 0 (0.0) | 0 (0.0) |  | 1 (3.6) | 1 (3.3) |  | 0 (0.0) | 0 (0.0) |  |
| TB |  |  |  |  |  |  |  |  |  |
| Pulmonary | 35 (97.2) | 37 (97.4) | >0.999 | 25 (89.3) | 29 (96.7) | 0.344 | 16 (100.0) | 18 (100.0) | -- |
| Extra-pulmonary | 1 (2.8) | 1 (2.6) |  | 3 (10.7) | 1 (3.3) |  | 0 (0.0) | 0 (0.0) |  |
| Bacilloscopy |  |  |  |  |  |  |  |  |  |
| No | 5 (13.5) | 9 (23.1) | 0.282 | 3 (10.7) | 9 (30.0) | 0.070 | 1 (6.2) | 0 (0.0) | 0.471 |
| Yes | 32 (86.5) | 30 (76.9) |  | 25 (89.3) | 21 (70.0) |  | 15 (93.8) | 18 (100.0) |  |
| HIV Test |  |  |  |  |  |  |  |  |  |
| No | 9 (24.3) | 10 (25.6) | 0.895 | 4 (14.3) | 7 (23.3) | 0.380 | 4 (25.0) | 7 (38.9) | 0.477 |
| Yes | 28 (75.7) | 29 (74.4) |  | 24 (85.7) | 23 (76.7) |  | 12 (75.0) | 11 (61.1) |  |

**Protocolo de estudo para avaliar o impacto da pandemia da SRA-CoV-2 no acesso aos serviços de saúde em Angola, com enfoque na prevenção e tratamento da tuberculose**

**Ficha informativa para trabalhadores da saúde**

| **Informação geral** | |
| --- | --- |
| **1. Código** \|__\|\|\|\|\| **Código** | **2. Data da Entrevista** \|\|\|/\|\|__\|/\|__\|\|\|\|\|\| |
| **3. Unidade de saúde de afiliação** |  |
| [1] Dispensário Anti Tubercolise e Lepra (DATL) | [5] Centro de Saúde Preventorio Infantil |
| [2] Hospital Divina Providência | [6] Centro de Saúde Cassequel |
| [3] Hospital Municipal De Kilamba Kiaxi | [7] Centro de Saúde Samba |
| [4] Hospital Municipal De Talatona | [8] Centro de Saúde Boavista |
| **4. Departamento** |  |
| [1] Fisiologia | [8] Nutrição |
| [2] Laboratório de baciloscopia | [9] Aconselhamento sobre o VIH |
| [3] Primeiros socorros | [10] PAV (vacinação infantil) |
| [4] Consulta externa | [11] Psicologia |
| [5] Medicina | [12] Oficina geral |
| [6] Consulta pré-natal | [13] Farmácia |
| [7] Cuidados infantis |  |
| **5. Nome Inicial\|\|\|__\|\|\|** | **6. Apelido inicial\|__\|\|\| 6.** |
| **7. Género** | **8. Idade** |
| [1] Macho [2] Fêmea | \|__\|__\| |
| **9. Qualificação** |  |
| [1] médico | [4] "Estaticista" |
| [2] enfermeira | [5] outros |
| [3] técnico de laboratório | specificare _________________________________ |
|  |  |
| **Impacto da Covid-19** | |
| Situação pandémica | |
| **10. Os dados internacionais descrevem Angola como um país pouco afectado pela pandemia, na sua opinião isto é atribuível a Angola: (resposta múltipla)** | |
| [1] Regulamentos muito rigorosos adoptados antecipadamente pelas autoridades | |
| [2] Subnotificação de casos | |
| [3] A idade jovem da população | |
| [4] O clima quente | |
| [5] Hábito de reunião principalmente ao ar livre | |
| [6] Outros, por favor especifique _______________________________________________________ | |
|  | |
| **11. Na sua opinião, a verdadeira propagação da pandemia em Angola foi:** | |
| [1] Comparável com o que é relatado no resto do mundo | |
| [2] Comparável com o resto do continente | |
| [3] A pandemia afectou Angola de forma mais branda do que outros países africanos | |
| [4] A pandemia afectou Angola mais do que outros países africanos | |
|  | |
| Impacto nos doentes | |
| **Durante o período pandémico:** | |
|  | |
| **12. Tem notado uma diminuição do número de doentes no serviço onde trabalha?** | |
| [1] Sim [0] Não [9] Não sei | |
| Em caso afirmativo, **os pacientes que se apresentaram tinham, em média, condições mais graves do que antes da pandemia (em comparação com a sua condição clínica de base)?** | |
| [1] Sim [0] Não [9] Não sei | |
|  | |
| **Na sua opinião, qual foi a razão para a diminuição do número de doentes (resposta múltipla)** | |
| [1] Problemas para chegar às instalações devido à falta de transportes públicos | |
| [2] Medo de ser testado para o Sars-CoV-2 e de ser fechado em instalações especiais no caso de um resultado positivo no teste | |
| [3] Medo de contrair o vírus | |
| [4] O agravamento da situação económica dificultou o acesso à unidade de saúde | |
| [5] Consciência do número decrescente de pessoal de saúde disponível, o que levou à limitação dos serviços | |
| [6] Sensibilização para a falta de tratamentos necessários para diferentes condições clínicas | |
| [7] Redução do horário de funcionamento dos estabelecimentos de saúde | |
|  | |
| **A diminuição da assistência afectou todos os grupos etários?** | |
| [1] Sim [0] Não [9] Não sei | |
| Caso contrário, **qual foi a banda em que notou uma diminuição maior?** | |
| [1] Crianças pequenas | |
| [2] Adolescentes/jovens | |
| [3] Adultos | |
| **A diminuição da afluência às urnas afectou igualmente ambos os sexos?** | |
| [1] Sim | |
| [2] Não, a redução foi maior nas mulheres | |
| [3] Não, a redução foi maior nos homens | |
|  | |
| Impacto nos trabalhadores da saúde | |
| **Durante o período pandémico:** | |
|  | |
| **13. Houve alguma alteração no número de pessoal de saúde empregado?** | |
| [1] Sim [0] Não [9] Não sei | |
| Em caso afirmativo, **em que direcção?** | |
| [1] diminuiu para reduzir o risco de contágio [2] aumentou para fazer face à emergência | |
|  | |
| **14. Houve alguma alteração nas listas de pessoal?** | |
| [1] Sim [0] Não [9] Não sei | |
| Em caso afirmativo, **como?** | |
| [1] turnos mais longos [2] turnos mais curtos | |
|  | |
| **15. O horário de abertura ao público foi limitado em comparação com o normal?** | |
| [1] Sim [0] Não [9] Não sei | |
|  | |
| **16. Pode quantificar de 1 a 10 quanto é que a pandemia mudou de trabalho?** | |
| [1][2][3][4] [5][6][7] [8] [9] [10] | |
|  | |
| **17. Havia algum método de compensação pelo aumento da carga de trabalho?** | |
| [1] Sim [0] Não [9] Não sei | |
| Em caso afirmativo, **em que direcção?** | |
| [1] aumento salarial [2] outro, por favor especifique ........... | |
|  | |
| **18. Em 2022, os turnos de trabalho estão a voltar ao normal?** | |
| [1] Sim [0] Não [9] Não sei | |
|  | |
| Utilização de equipamento de protecção pessoal | |
| **19. O equipamento de protecção pessoal estava adequadamente disponível nas instalações?** | |
| [1] Sim [0] Não [9] Não sei | |
|  | |
| **20. A necessidade de cumprir os regulamentos de segurança levou a dificuldades na realização das próprias actividades?** | |
| [1] Sim [0] Não [9] Não sei | |
|  | |
| **21. As regras de segurança foram respeitadas pelo pessoal?** | |
| [1] Sim [0] Não [2] Apenas parcialmente [9] Não sei | |

**Protocolo de estudo para avaliar o impacto da pandemia da SRA-CoV-2 no acesso aos serviços de saúde em Angola, com enfoque na prevenção e tratamento da tuberculose**

**Cartão do paciente**

| **Identificadores e dados gerais do registo de doentes** | |
| --- | --- |
| **1. Mês/ano de referência** \|__\|\|/\|\|__\|\|\|\| | **Data da Entrevista** \|\|\|/\|__\|/\|__\|\|\|\|\| |
|  |  |
| **2. Código no registo** \|__\|\|\|/\|\|\|\|\| | **3. Nome do entrevistador _________________________** |
|  |  |
| **4. Estrutura de referência sanitária** |  |
| [1] Dispensário Anti Tubercolise e Lepra (DATL) | [5] Centro de Saúde Preventorio Infantil |
| [2] Hospital Divina Providência | [6] Centro de Saúde Cassequel |
| [3] Hospital Municipal De Kilamba Kiaxi | [7] Centro de Saúde Samba |
| [4] Hospital Municipal De Talatona | [8] Centro de Saúde Boavista |
|  |  |
| **5. Género** | **6. Idade** |
| [1] Macho [2] Fêmea | \|__\|__\| |
|  |  |
| **7. Classificação de casos** | **8. Localização** |
| [1] novo | [1] Pulmão |
| [2] já tratado | [2] Extrapulmonar |
| [3] transferido |  |
|  |  |
| **9. Baciloscopia** | **10. Se realizada, a Baciloscopia resulta** |
| [1] realizado | [1] Positivo |
| [0] não executado | [0] Negativo |
|  |  |
| **11. Testagem do VIH** | **12. Se realizado, resultado do teste VIH** |
| [1] realizado | [1] Positivo |
| [0] não executado | [0] Negativo |
|  |  |
| **13. Resultado do tratamento** |  |
| [1] Curado | [4] Perda de vista de |
| [2] Tratamento terminado | [5] Não avaliado |
| [3] Falha terapêutica |  |

| **Entrevista com o paciente (para as perguntas 18 a 24, consulte o período de referência do processo médico seleccionado)** | |
| --- | --- |
| **14. Nível de educação** |  |
| [1] Não alfabetizato | [4] Ensino Secundário. 2º Ciclo |
| [2] Ensino Primário | [5] Universidade |
| [3] Ensino Secundário. 1º Ciclo |  |
|  |  |
| **15. Profissão** |  |
| [1] Desempregado | [5] Camponês |
| [2] Trabalho ocasional | [6] Estudante |
| [3] Varejo | [7] Outros |
| [4] Funcionário do governo | Outro, especifique por favor ______________________________ |
|  |  |
| **16. Municipio di residenza** _____________________________________________________________________ | |
|  |  |
| **17. Nº de coabitantes** | \|__\|__\| |
|  | |
| **18. Havia uma pessoa de referência na família que o apoiasse no 'lidar com a doença'?** | |
| [1] Sim [0] Não | |
|  | |
| **19. A que distância está a sua casa do estabelecimento de saúde que o atendeu?** | |
| \|__\|\| Km | |
|  | |
| **20. Que meio de transporte utilizou para se deslocar ao estabelecimento de saúde?** | |
| ______________________________________________________________ | |
|  | |
| **21. No caminho de casa para o estabelecimento de saúde, estava preocupado em adquirir uma infecção no meio de transporte?** | |
| [1] Sim [0] Não | **Em caso afirmativo, qual?** _________________________________ |
|  | |
| **22. Tinha preocupações sobre a aquisição de uma infecção dentro do estabelecimento de saúde?** | |
| [1] Sim [0] Não | **Em caso afirmativo, qual?** _________________________________ |
|  | |
| **23. Durante a sua doença, alguma vez teve de utilizar farmácias privadas para comprar medicamentos?** | |
| [1] Sim [0] Não | |
|  | |
| **24. Para além do episódio de tuberculose a que agora nos referimos, por acaso foi novamente seguido por uma unidade sanitária de tuberculose?** | |
| [1] Sim [0] Não | **Em caso afirmativo, em que ano?** \|\|\|\|\|\| |
|  | **Em caso afirmativo, em que estrutura** ____________________________ |
|  | |
|  | |
| **Secção Covid-19: candidaturas para pacientes inscritos nos anos 2020-2022** | |
|  | |
| **Durante a pandemia:** | |
|  | |
| **25. Teve problemas para chegar ao estabelecimento de saúde devido à falta de transportes públicos?** | |
| [Sim, sempre [2] Sim, por vezes [3] Raramente [4] Nunca [9] Não sei | |
|  | |
| **26. Tinha medo de ser testado para o Sars-CoV-2?** | |
| [Sim, sempre [2] Sim, por vezes [3] Raramente [4] Nunca [9] Não sei | |
|  | |
| **26. Teve medo de testar positivo para Sars-CoV-2 e de ser colocado em instalações especiais?** | |
| [Sim, sempre [2] Sim, por vezes [3] Raramente [4] Nunca [9] Não sei | |
|  | |
| **Temeu o isolamento social como resultado de uma possível infecção por Sars-CoV-2?** | |
| [Sim, sempre [2] Sim, por vezes [3] Raramente [4] Nunca [9] Não sei | |
|  | |
| **Aconteceu-te que não foste às instalações que frequentavas sabendo que o pessoal estaria ausente?** | |
| [Sim, sempre [2] Sim, por vezes [3] Raramente [4] Nunca [9] Não sei | |
|  | |
| **Aconteceu-te que não foste às instalações que te estavam a tratar sabendo que os medicamentos não estavam disponíveis?** | |
| [Sim, sempre [2] Sim, por vezes [3] Raramente [4] Nunca [9] Não sei | |
|  | |
| **Sentiu uma redução nos seus rendimentos devido à pandemia?** | |
| [Sim, sempre [2] Sim, por vezes [3] Raramente [4] Nunca [9] Não sei | |
|  | |
